# Compounding endowment effects when trading draft picks in the Australian Football League

**DOI:** 10.1371/journal.pone.0300546

**Published:** 2024-03-14

**Authors:** Jemuel Chandrakumaran, Paul Larkin, Sam McIntosh, Sam Robertson

**Affiliations:** 1 Institute for Health and Sport (IHES), Victoria University, Melbourne, Australia; 2 MSA Research Centre, Maribyrnong Sports Academy, Melbourne, Australia; 3 Western Bulldogs Football Club, Melbourne, Australia; Deakin University, AUSTRALIA

## Abstract

Endowment effect relates to a situation when decision makers are more likely to retain an object they own, than acquire the same object when they do not own it. Studies have often concluded that players recruited early on through drafts are more likely to be held in team rosters irrespective of their marginal utility. We tested the hypothesis wherein this effect would compound when the pick used to select a player is traded between teams. Using a sample of draftees selected between 2003 and 2016 in the Australian Football League, we created a proportional hazard model to predict the career longevity of a player with their drafting team and overall career. The results suggest each subsequent trade marginally reduced the exit of a player by a log normal rate of 0.269 in their career with the team that initially drafted them. The findings were attributed to the premium requested by the original team that is compounded with every exchange as the reference points used to determine value have also shifted with the trade.

## Introduction

Player drafts are commonly used as a labour market intervention technique to equitably allocate amateur talent in sport. Most leagues practice a reverse order system (or a derivate of the same) whereby the team that finished last in the season immediately before the draft gets awarded the first pick followed by the second last [1]. After all competing teams make their respective choices (also called a round), the process is repeated. The principle governing the draft aims to cyclically alter the fortunes of each team in the league assuming they all intend to maximise their wins [2]. Though some teams might aim to maximise profits, maximising wins will correlate to this objective through fan engagement, commercial partnerships, and brand value appreciation. The picks themselves essentially serve as financial assets that teams could realize either by selecting a player from the talent pool or exchanging them for other picks (both in the current draft or the future) and players or a combination of both. Whilst this allows teams to efficiently exchange selections between themselves, studies have shown teams in the National Football League (NFL) tend to overvalue the picks that are thus endowed upon them when comparing draftee payments and expected outcomes [3]. This inherently leads to a behaviour known as ‘escalation of commitment’, whereby decision-makers maintain (or increase) their commitment to a decision they made in the past, even when the marginal cost outweighs the marginal benefit [4].

In the professional sporting context, one could assume any win-maximising team would only retain players in their roster that could effectively contribute to this shared goal. Some might retain players for their brand value, though in the long-term teams will have to weigh in the opportunity cost of holding on to so such players in lieu of making a concentrated effort towards building a championship winning team. However, Staw and Hoang [5] contradicted this predisposition finding teams within the National Basketball Association (NBA) gave more playing time and are more likely to retain players selected early on in the player draft, irrespective of their marginal productivity. The authors coined this behaviour as ‘sunk investment play’, given decision-makers throw good money after bad to justify the initial sunk cost of drafting the player (similar to the disposition effect in behavioural finance literature [6]). Researchers have reaffirmed this phenomenon in the NBA [7–9] and other sporting competitions such as the NFL [10,11], National Hockey League (NHL, [12]) whilst Borland et al [13] showed minimal effects in the Australian Football League (AFL). However, Chandrakumaran [14] concluded that this effect might prevail in the AFL as the difference between inferred and actual value was lesser for early picks (similar to the NFL [3]). This was attributed to the overutilisation of players selected early on in the draft to justify the initial choice. Still, one could question if similar behaviours were exhibited when such players were obtained through traded picks. Furthermore, if the same pick was traded multiple times before it was exercised to choose a player, would the previous behaviour remain the same.

The existing literature in behavioural economics provides some foundation in conceptualising the decision-making process with uncertain prospective payoffs. As per the expected utility theory, a rational agent would choose the best option by comparing the expected utility of each outcome [15]. However, upon evaluating the numerous behavioural biases leading to sub-optimal decision-making, Kahneman and Tversky [16] concluded that agents are generally loss averse (not risk averse) and feel the impact of losses greater than an equivalent gain (commonly referred to as prospect theory). Their theory suggested agents generally comprehend these gains and losses compared to a reference point that is subjective to each individual and situation, causing their decisions to be made in relativity and not in absolutes. This inherently creates a situation called the endowment effect, whereby agents are highly likely to retain the ownership of an object they would not otherwise own. Studies have attributed such behaviours to under weighing the opportunity cost of possessing it [17] caused by the psychological inertia governing their initial purchase decision [18]. In such situations, if an owner is inclined to sell the object, the price at which they are willing to part ways is usually higher than the price prospective buyers are disposed to offer [19]. Amidst such disequilibrium, should a sale happen, the buyer’s reference point for the gain-loss asymmetry will shift relative to the existing point or status quo [20,21] and we would expect that effect to be compounded across every subsequent chain event [22].

**Fig 1:**
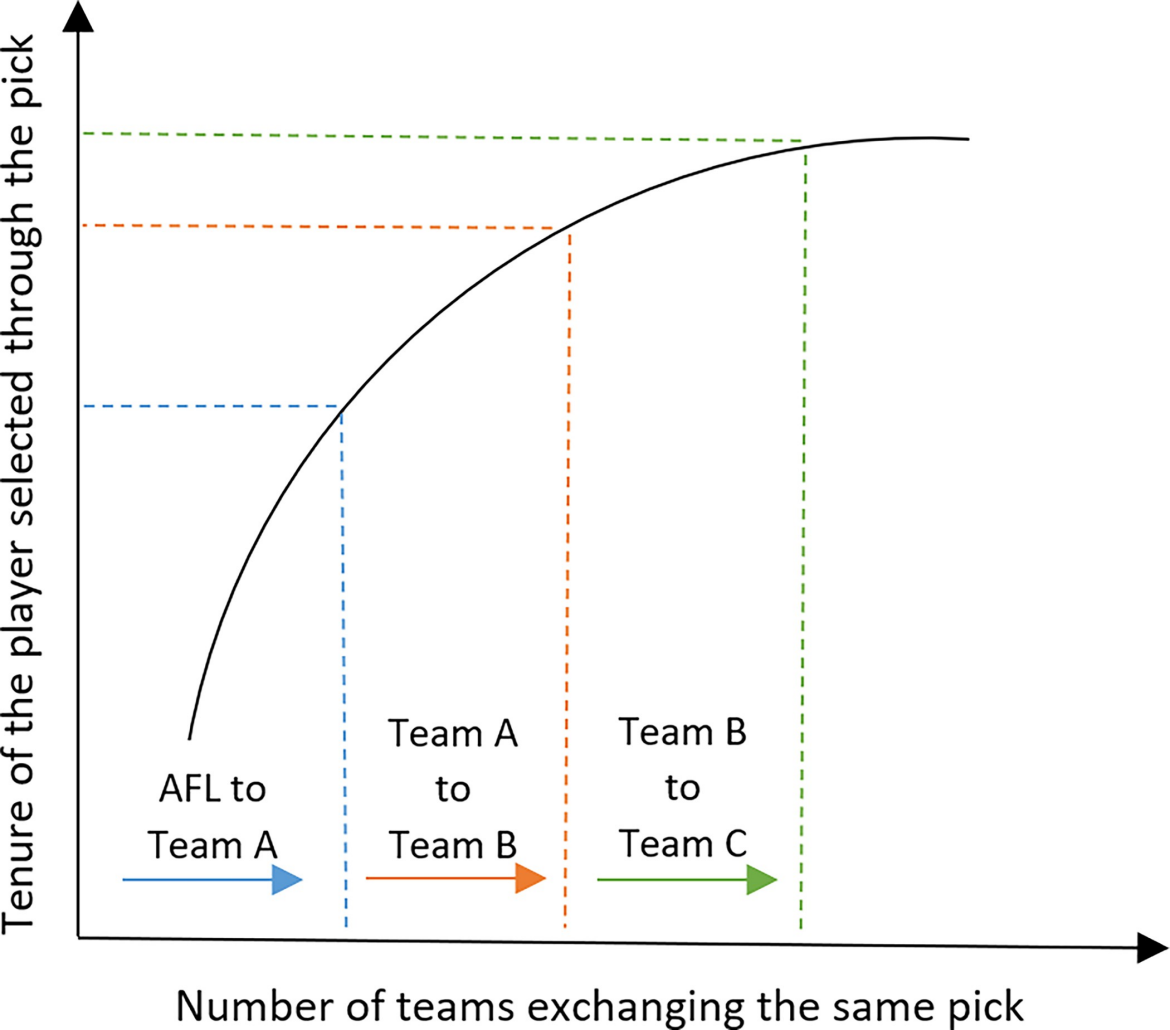
Conceptual model of the hypothesis.

Should the same behaviour be observed within the pick trading market, when a team is allocated picks in the draft based on a pre-approved system, the reference point used by teams to adjudge trades could be higher, showcasing the endowment effect. A previous study explored this phenomenon within major sports drafts in North America and found strong evidence of this effect whereby teams would recoup the original picks which were appropriated to them by the league, though they may have previously exchanged them [23]. However, if the initial team to whom the picks were initially endowed by the league choose to trade the picks, the effect will be compounded on players selected through traded picks with each subsequent exchange as the seller’s premium increases as shown in Fig 1 (where draftee tenure is used as a proxy to quantify the endowment effect). That is to say, if team B trades up and purchases a pick from team A in exchange for a group of B’s current picks, and team B uses the pick to select a player, team B will hold the player irrespective of their marginal productivity longer than team A. This is because the value demanded by team A to release a pick it was originally endowed by the league to team B would be higher. Hence team B will need to justify the excess and thereby hold the player longer. Building on this, should team C trade the same pick from team B that was originally obtained from team A, team C will hold any player they select using that pick longer than either teams A or B, had they exercised the pick themselves. This paper thereby aims to test the hypothesis wherein the endowment effect would compound when the pick used to select a player is traded between teams.

## Data & methods

In order to evaluate this hypothesis, we used the case of the AFL player draft due to both its uniqueness as a sport and its effectiveness in the trade market. Unlike the North American sporting codes, the AFL has a smaller draft (NFL and NHL draft has in excess of 230 picks, whereas the AFL draft averages less than a 100) and requires teams to maintain a smaller roster (NFL allows a 90-man list in the off season which could be weaned to 53 at the start of the season) indirectly achieving rank order selection parity [14]. This ensures all agents effectively evaluate potential recruits minimising the effect of alternative motives on their choice. Furthermore, as Australian rules football has 18 players per team in the field of play, the effect a single player would have on the outcome of a contest will not be significant, unlike the NBA, making the list of potential recruits largely homogenous [14]. Teams are awarded selections based on their reverse order season standing with the club finishing last having the first pick, followed by the second last. After each team (currently 18 teams) successfully selects a player, the process is repeated approximately three more times. The order of selection can be influenced by a variety of other rules, such as free agency compensation (when teams are awarded additional picks when there is a net outflow of veteran players in the trade period) and priority picks (when the league awards additional picks to support teams which consistently perform below par). In addition to this, teams are also allowed to trade picks with other clubs, for pick(s) in the current year or one year into the future, active players, or a combination of both. However, until recently, a majority of picks were traded for players. Players selected through the draft are usually signed on for two seasons [24]. At the end of year two, the player will be restricted from moving to another team unless the team delists him. If the player is re-signed by the team, they can continue playing with their original team. Otherwise, the player can solicit offers from other clubs or re-enter the draft. It is important to note that player salary and contract information is not made publicly available in the AFL.

Data on all AFL draftees selected between 2003 and 2016 (refer to S1 File) was obtained from Sorensen Technologies (a third-party data agency that provides data for the gambling industry sourced from the AFL) together with their subsequent performance from 2004 to 2017 (this was cross referenced to other open-source sites such as www.afltables.com). Trade related data was compiled from www.draftguru.com.au. Also, as some non-amateur players are selected at points not consistent with their post draft performance, rookie elevations (teams wishing to move players from their rookie list to the seniors will use picks later on in the draft which may not accurately reflect their profiles) and players who have played before (if a player is delisted from a team and isn’t able to secure a position with another club in the trading period, they are allowed to enter the draft talent pool), were excluded from the sample (as done in previous studies [25]). In addition, those selected after pick 73 were also removed from the analysis to ensure a uniform end point similar to the AFL’s draft value index (DVI, The DVI was introduced in 2015 by the league to facilitate the Father-Son and Club-Academy rules by appropriating a numerical value for each selection in the draft. The index was created by fitting the career compensation of draftees selected over fifteen years [26]). This yielded a sample of 905 draftees.

Table 1 shows the exchange rate of all picks within the sample. In total, 37% of all draft picks changed hands prior to the selection of a player, of which each pick was traded 1.49 times on average. 218 of these were traded once, 88 twice, and 29 three or more times. The breakdown also shows picks between 31 and 60 were on average traded more often. As the top five selections seldom moved, it reinforces findings from previous studies which have shown the correlation of endowment effects to such early picks [27].

**Table 1:** Trade summary of all draft picks within the sample.

Description	No. of Picks	No. of All Traded Picks	Avg. Times Traded	% Of Traded Picks	No. of Traded Picks per Times Exchanged		
					1	2	≥3
Total	905	335	1.49	37.02%	218	88	29
Picks							
1 to 5	70	9	1.11	12.86%	8	1	-
6 to 15	140	44	1.27	31.43%	36	6	2
16 to 30	209	81	1.46	38.76%	49	28	4
31 to 45	203	88	1.73	43.35%	46	30	12
46 to 60	180	77	1.52	42.78%	53	14	10
61 to 73	103	36	1.31	34.95%	26	9	1

**Fig 2:**
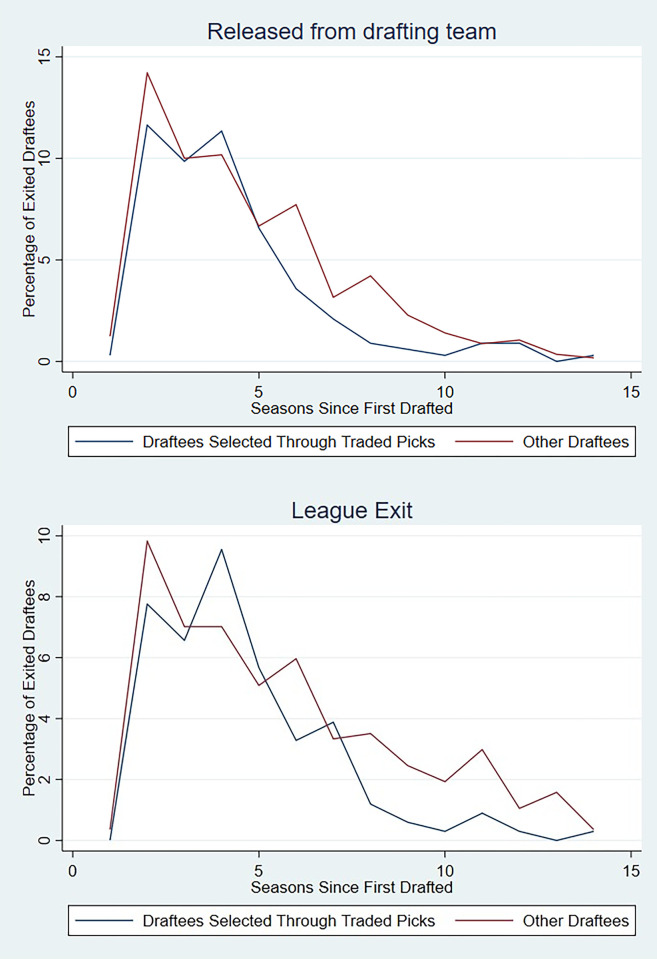
Percentage of players released by their drafting teams and exiting the league each year post draft.

Fig 2 shows the proportion of players who exit from the team that drafted them and their career respectively plotted against their experience. Both show a negatively skewed relationship demonstrating the behaviour of teams removing players after the expiry of their initial contracts [28]. The figures also show that in most years, players selected through traded picks are less likely to exit both the team that originally drafted them and the league (Fig 2, note: 52% of the draftees observed within the sample were still actively playing in the league as of 2017). This observation reconciles with the hypothesis, whereby players selected through traded picks tend to last longer within the league.

Table 2 shows the difference in draftee career tenure based on the number of times the picks used to select them were traded beforehand. The multivariate test of means shows significant variations in career length between those selected using traded and non-traded picks. However, the means themselves suggest smaller career lengths for those selected through traded picks, which is contrary to the proposed hypothesis. Yet, as shown in Table 1, most picks which are traded seem to be situated later on in the draft, the corresponding career tenures of players selected through such picks might be comparatively low. This is also observable in the average pick number of the selections exchanged displayed in Table 2. The average pick number used by teams exercising their own picks stood at 32, whilst picks exchanged once or twice averaged to 36. Finally, the t-test results for variances of career tenures between draftees selected using non-traded picks and picks which were traded once shows significant variation, similar to those traded more than once. However, their significance reduces when comparing draftees selected using picks traded once and twice or twice and thrice.

**Table 2:** Mean analysis within groups of traded picks.

Mean Variable				Career Length with Drafting Team		Total Career Length	
Multivariate tests of means between traded and non-traded picks							
	Wils’ Lambda			0.959***		0.936***	
	Pillai’s Trace			0.041***		0.064***	
	Lawley-Hotelling Trace			0.043***		0.068***	
	Roy’s Largest Root			0.043***		0.068***	
							
Times Traded	Avg. Pick	Obs		Mean	Std. Err.	Mean	Std. Err.
0	32.044	570		4.974	0.122	5.909	0.141
1	36.032	218		4.092	0.181	4.564	0.191
2	36.534	88		3.659	0.268	4.068	0.306
≥3	39.828	29		2.966	0.487	3.138	0.524
							
Within Group (Times Traded)		Degrees of Freedom		T-stat		T-stat	
0 & 1		786		3.879***		5.241***	
0 & 2		656		3.994***		4.870***	
0 & ≥3		597		3.624***		4.361***	
1 & 2		304		1.305*		1.384*	
1 & ≥3		245		2.141**		2.554***	
2 & ≥3		115		1.273		1.520*	

*** p<0.01

** p<0.05

* p<0.1.

Given the research question aimed to be answered here looks at the difference in career lengths based on the nature of the picks used to select them it is important to control the data to account for active players as they might not reach the failure event. Hence, the sample was defined as a survival data series setting the longevity of players (in number of seasons) as the time variable and a binary variable with a value of 1 for those who have been delisted or retired and 0 for those still active in the league as the failure event. This was used to generate Table 3 which displays the incident rates (number of failure events divided by the total time) stratified by picks groups and the number of times the picks were traded. Ideally a lower incident rate would be preferred by draftees as it means more players do not reach the failure event leading longer careers within their teams. When looking at the career prospects of a player selected within picks 31 and 45, the incident rates decrease as pick used to draft player exchanges more hands. The same cannot be inferred from early picks (6 to 15). This reconciles with the previous assertion alluded to in Table 2, where the mean career tenures of players selected through traded picks were lower than those selected through non-traded picks as most trades happen later on in the draft.

**Table 3:** Incident rates of draftees selected at various points in the draft based on the number of times their picks were traded.

Picks		No. of Times Picks were Traded			
		0	1	2	≥3
1 to 5	Career with Drafting Team	0.066	0.107	0.000	-
6 to 15		0.100	0.091	0.143	0.167
16 to 30		0.113	0.116	0.101	0.042
31 to 45		0.141	0.143	0.095	0.094
46 to 60		0.167	0.207	0.125	0.182
61 to 73		0.224	0.141	0.053	0.000
1 to 5	Total Career	0.028	0.000	0.000	-
6 to 15		0.051	0.045	0.107	0.111
16 to 30		0.078	0.073	0.087	0.042
31 to 45		0.100	0.116	0.063	0.088
46 to 60		0.136	0.179	0.097	0.182
61 to 73		0.189	0.120	0.000	0.000

However, pick number alone cannot be used to evaluate the prospect of a player, as a player’s utility to a team would be a product of many competing factors, such as position played, physical metrics, on-field performance, and team requirements. In order to verify this theory, two Cox proportional hazard models were estimated. Unlike a conventional binary model (logit or probit), the Cox proportional hazards model would easily differentiate past and current players based on their career lengths (which is a proxy for the year in which the player was selected). The explanatory variables included the pick number used to select the player. The contribution to margin of victory (CMV) in the team that drafted them and in their overall career was added as a performance variable to justify the retention of a player across multiple seasons. The CMV metric was obtained by regressing the difference of a set of on-field metrics such as kicks and marks, which translated to point differences in each regular season AFL game between 2003 and 2017, similar to comparable models prevalent in the literature [29–31] (it is worthwhile noting that AFL’s official player rating system (Champion Data Player Ratings) was also developed using a similar method). After refining the predictors using a stepwise analysis accounting for the Bayesian Information Criterion (BIC), the coefficients were used to extrapolate the career and seasonal CMV per draftee used in this study both in their overall career and their career with the team which initially drafted them. Coincidentally, previous studies which have used CMV to evaluate draft outcomes showed no significant difference between players selected through traded and non-traded picks [14], suggesting career tenures to also be the same in a rational setting. However, we chose to interact this term with the position played (though there are multiple positions in Australian rules football, this study uses the main four groups which are defenders, forwards, midfielders, and ruckmen) by the player as the opportunity for players to create scoring stats is not evenly distributed across all positions. Initial iterations of the model used games played or time on field (in minutes) instead of CMV. However, with previous studies suggesting the decisional biases behind the use of players on field and their ineffectiveness in predicting performance, the CMV variable was used in the final model [32,33]. The list was further compensated by indicators for race (indigenous players recruited through the draft have known to outperform their counterparts [34]), Father-Son (F/S) selections prior to 2007 (teams who have selected sons of former players prior to 2007 were required to nominate the player using a third round draft pick which did not always correlate to their expected outcomes [35]), and drafting team. The primary count variable for this study, number of owners, was used to show how many teams had the selection before a player was ultimately chosen. All picks will have a value of one by default, unless they were traded, where it will be replaced by two or more based on the number times they were exchanged. The natural log of this count was used to show the increase in endowment effect with each trade at a diminished rate [36]. For the hypothesis to hold the coefficient of the owner variable should yield a negative value.

## Results

As per the results shown in Table 4, all the control variables used in the models concurred with the existing literature. The positive coefficient attracted by the pick (the pick variable was tested in alternative forms such a quadratic and natural log yielding similar results) suggested a direct monotonic relationship between pick and career length as players selected early on in the draft would last longer compared to their counterparts [5]. F/S recruits prior to 2007 remained in par with their counterparts suggesting that whilst their pick number might not represent post draft outcomes, their tenure within the league might not be affected by it. Indigenous players did have a shorter career than their peers. This might not necessarily be due to underperformance, as studies have shown such players to be more productive [34], but rather due to the longer transition time in assimilating indigenous players within the league [37–39]. On the other hand, when draftees effectively contribute to the team as represented by their CMV, it reduces their chances of leaving. Midfielders and forwards reduced the chances of exiting the drafting team by 0.002 per CMV, whilst defenders and ruckmen did so by 0.003 and 0.004 per CMV respectively. The slightly higher values attributed to defenders and ruckmen was due to the inability of such positions to effectively contribute towards scoring outcomes unlike forwards and midfielders [40]. However, since a player’s contribution to a team’s chances of winning generally follows a normal distribution (similar to a bell curve) over their overall tenure [25], an indirect linear relationship between CMV and tenure could not be inferred. After peaking at a certain point in their career, veteran players would either resign or be replaced by teams to ensure continuance. Interestingly when both games played and time on field (in minutes) was used instead of CMV, a similar outcome was observed. Apart from Geelong, most drafting teams remained neutral in the first model, but yielded positive results on the career model.

**Table 4:** Regression results for the endowment effect model.

Model Type		Cox Proportional Hazard Model				
Dependent Variable		Career Length with Drafting Team			Total Career Length	
Model Number		(1)			(2)	
Independent Variables		Coefficient	Std. Err.		Coefficient	Std. Err.
Pick		0.005*	0.002		0.012***	0.003
Indigenous		0.352**	0.168		0.367**	0.183
F/S <2007		0.291	0.278		0.316	0.301
***ln(No*. *of Owners)***		***-0*.*269*** **	***0*.*113***		***-0*.*076***	***0*.*122***
Interaction of CMV acquired whilst employed by the Drafting Team with Player Position						
Defender		-0.003***	0.002			
Forward		-0.002***	0.001			
Midfielder		-0.002***	0.001			
Ruckman		-0.004***	0.001			
Interaction of CMV acquired across total career with Player Position						
Defender					-0.003***	0.002
Forward					-0.002***	0.002
Midfielder					-0.002***	0.001
Ruckman					-0.005***	0.001
Drafting Team ^a^						
Brisbane		0.284	0.240		0.480*	0.281
Carlton		0.359	0.248		0.945***	0.279
Collingwood		0.105	0.256		0.536*	0.293
Essendon		0.020	0.252		0.722**	0.284
Fremantle		0.037	0.254		0.810***	0.284
Geelong		-0.582**	0.262		-0.238	0.301
Gold Coast		0.071	0.358		0.484	0.421
Greater Western Sydney		0.333	0.318		-0.718	0.623
Hawthorn		-0.354	0.265		-0.335	0.317
Melbourne		0.183	0.250		0.678**	0.286
North Melbourne		-0.058	0.261		0.555*	0.293
Port Adelaide		-0.394	0.266		0.342	0.292
Richmond		-0.035	0.254		0.459	0.283
St. Kilda		0.084	0.283		0.721**	0.323
Sydney		-0.055	0.262		-0.226	0.303
West Coast		-0.396	0.288		0.242	0.314
Western Bulldogs		-0.191	0.261		0.132	0.299
No. of Players		905			905	
No. of Failures		526			433	
Log likelihood		-2831.784			-2224.600	
LR Chi^2^		621.870			704.470	
Prob > Chi^2^		0.000			0.000	

^a^ Reference team is Adelaide.

*** p<0.01

** p<0.05

* p<0.1.

The key finding of this model is that a player’s longevity with their drafting team, significantly reduces with every trade (or new owner) at a diminishing log normal rate of -0.269 (significant at 0.05). Interestingly when the same variable was set as an indicator variable, the individual coefficients for each step followed a log normal increase as well. This corresponds with the hypothesis that a team would retain a player it selects through the draft if the pick used to select the player was acquired through a trade. However, the same effect wasn’t observable across the player’s career. When such drafted players eventually move to different teams in their career, this effect could be minimised as the initial investment into the player went only with the team that drafted them and not the subsequent teams that the player moved on to. On a practical standpoint, managers should hold a player in their lists based on their marginal productivity (both on and off field). However, when the initial investment to trade up (i.e., procuring the pick) has a higher cost base, the reluctance on the decision maker’s end on riding the option is understandable. In order to avoid such a dilemma, the best course of action would be to ensure all trades are made in alignment with their club’s valuation of each pick, as the subsequent mentality to hold is a product of the first (especially when the same pick has changed hands before).

## Discussion

The endowment effect is a decisional irrationality that exists in all facets of human life [4]. When applied directly to the player draft, existing literature within the NBA has shown how decision-makers retain drafted players irrespective of their marginal productivity in order to validate their previous resolve [5]. This effect was further evident amongst players drafted early on the system as they would have a higher level of emotional investment [3,14]. The lower number of trades observed in the first five picks further shows the prevalence of the endowment effect as teams were unwilling to deal on trades including these selections. The findings from this study fails to falsify the theory upholding the proposed hypothesis. Interestingly, this effect still manifests even amongst teams that only acquired the right for the pick.

Kahneman et al [19] suggested the endowment effect might prevail even if there is no cause for attachment (or recently obtained/purchased). Furthermore, when a pick is traded, the value demanded by the original owner is usually higher. This results in a situation where the reference point of the subsequent owner moves further to the right (on a cartesian plane) as it has to account for the premium exchanged to acquire the pick. This could cause teams to inherently increase the value of the pick they recently acquired (if they intend to re-trade it) or retain any player they obtain through the pick for a longer period of time than the original owners might have. Whilst this behaviour might seem to exacerbate the endowment effect that already exists within the league, it is important to note this compounding anomaly is caused mainly due to the existence of the original effect.

While the results from this study have affirmed the hypothesis put forward, the model could be further improved. These could include modifications for changes in team decision makers (i.e., general managers and head coaches), injury lists and overall players salary cap management. Also, the timing between trades could play a part in the emotional attachment decision makers have. For example, a team that acquires a pick and trades it immediately might not necessarily demand a higher value in return when compared to another team which might have held the pick for a few days. Moreover, as the AFL started allowing teams to trade picks up to one year in the future [41], ‘trading up’ ought to be clearly defined as conventional wisdom suggests it to be the earliest pick in each trade. However, when teams trade into the future, an early selection from next year exchanged for a few late selections from today might misappropriate the notion behind the trade. Furthermore, though our work fails to falsify prospect theory, and by extension the endowment effect, it is important to note that agents behave more rationally as they gain experience within competitive markets [42]. Still, when buyers and sellers exchange goods and services at or close to the market price, the effect could be minimalised [43].

## Supporting information

S1 File Data used in the study. (XLSX)
